# In Vivo Stimulation of α- and β-Adrenoceptors in Mice Differentially Alters Small RNA Content of Circulating Extracellular Vesicles

**DOI:** 10.3390/cells10051211

**Published:** 2021-05-15

**Authors:** Jin-Sook Kwon, Eric W. Barr, J. Kurt Chuprun, Walter J. Koch

**Affiliations:** Center for Translational Medicine and Department of Pharmacology, Lewis Katz School of Medicine, Temple University, 3500 North Broad St, MERB 941, Philadelphia, PA 19140, USA; tue93116@temple.edu (J.-S.K.); eric.barr@temple.edu (E.W.B.); kurt.chuprun@temple.edu (J.K.C.)

**Keywords:** microRNA, extracellular vesicles (EVs), alpha-adrenergic receptors, beta-adrenergic receptors, blood, mouse

## Abstract

When myocardial function is compromised as in heart failure (HF), there is activation of the sympathetic nervous system with elevated circulating catecholamine levels. These catecholamines activate cardiac and extra-cardiac adrenergic receptors (ARs). Interest in secreted extracellular vesicles (EVs) from the heart is growing and in HF, it is not known whether excessive activation of α- or β-adrenergic receptors (ARs) could induce specific changes in EV content. In this study, we have evaluated, by next generation sequencing, the small RNA content, including micro-RNAs (miRs), of circulating EVs of mice exposed to chronic selective α- or β- AR stimulation. EVs from mouse blood were purified by differential ultracentrifugation resulting in EVs with an average size of 116.6 ± 4.8 nm that by immunoblotting included protein markers of EVs. We identified the presence of miRs in blood EVs using miR-21-5p and -16-5p real-time PCR as known constituents of blood exosomes that make up a portion of EVs. We next performed next generation sequencing (NGS) of small non-coding RNAs found in blood EVs from mice following 7 days of chronic treatment with isoproterenol (ISO) or phenylephrine (PE) to stimulate α- or β-ARs, respectively. PE increased the percent of genomic repeat region reads and decreased the percent of miR reads. In miR expression analysis, PE and ISO displayed specific patterns of miR expression that suggests differential pathway regulation. The top 20 KEGG pathways predicted by differential expressed miRs show that PE and ISO share 11 of 20 pathways analyzed and reveal also key differences including three synapse relative pathways induced by ISO relative to PE treatment. Both α-and β-AR agonists can alter small RNA content of circulating blood EVs/exosomes including differential expression and loading of miRs that indicate regulation of distinct pathways. This study provides novel insight into chronic sympathetic nervous system activation in HF where excessive catecholamines may not only participate in pathological remodeling of the heart but alter other organs due to secretion of EVs with altered miR content.

## 1. Introduction

Extracellular vesicles (EVs) released from cells [1] have been isolated from most cell types and also body fluids including plasma, saliva, pericardial fluid and urine [2]. EVs can be distinguished roughly on the basis of size: exosomes (50–150 nm), microvesicles (100–1000 nm), large oncosomes (1000–5000 nm) and apoptotic bodies (100–5000 nm) [3]. These different EVs are believed to contain cargo including proteins, lipids and also genetic components such as RNAs including non-coding RNAs (ncRNAs) that include microRNas (miRs) [1]. A prevailing hypothesis is that EVs carry this cargo for intra- and inter-organ communication including for normal homeostatic events such as cardiac development/growth, as well as pathology [1,4,5,6].

Growing evidence suggests that EVs may be used as diagnostic biomarkers or even carry therapeutic agents for cardiovascular diseases (CVDs) including ischemia/reperfusion injury, acute myocardial infarction (AMI), diabetic cardiomyopathy and septic cardiomyopathy [2,4,7,8]. For example, serum exosomes isolated from dilated cardiomyopathy (DCM) patients can mediate pathological responses in vitro in isolated cardiomyocytes [9]. Since DCM and other forms of heart failure (HF) are associated with activation of the sympathetic nervous system (SNS), there has been interest in how chronic activation of adrenergic receptors (ARs) may alter circulating EVs. Indeed, a study has shown that activation of α-ARs could modify the protein Hsp72 and miR-142-5p found in rat blood exosomes and these changes could be attenuated by α-AR blockade [10]. Due to heightened SNS activity in HF, myocardial β_1_-AR density is reduced [11] and these changes can correlate with disease severity [12]. Thus, knowing how chronic α- and β-AR activation may alter the content of circulating EVs, including exosomes, may shed light on how HF progresses and how the failing heart may influence distant organs.

The use of small animal models is a common way to investigate pathology of human disease, however, for circulating EV research, rodents can be problematic due to low blood volumes for EV isolation and quantities needed for a thorough investigation of content, and especially for an evaluation of ncRNA of exosomal content via next-generation sequencing (NGS). Therefore, only a few studies have reported exosomal RNA characterization by NGS using rodent samples [13,14], which remains challenging [15]. In this study, we used mice treated chronically with isoproterenol (ISO) or phenylephrine (PE) to stimulate α- or β-ARs, respectively, and determined ncRNA content of purified EVs from blood to determine whether SNS activation, as seen in HF, may influence EV content as a marker for contributing to CVD.

## 2. Materials and Methods

### 2.1. Isolation of Blood EVs in Mouse

Mouse blood EVs were isolated from blood serum as described previously with minor modification [16,17]. Briefly, under anesthesia using isoflurane, blood of mouse was collected by cutting the common carotid artery and was then placed at room temperature for 30 min for coagulation. After centrifugation at 13,000 rpm for 5 min, serum was collected. Immediately, the serum was diluted in 20 mL of phosphate-buffered saline (PBS) and followed with sequential centrifugation under 4 °C at 4400 rpm for 15 min (Optima L-90K ultracentrifuge; Beckman Coulter SW 70 Ti rotor), at 10,000× *g* for 30 min to remove cell debris, and 100,000× *g* for 2 h. The final supernatant was discarded and the EV pellet was used to check the morphology, to purify total RNA and to purify protein.

### 2.2. Basal Characterization of Mouse Blood Serum EVs

#### 2.2.1. Analysis of EV Morphology

We prepared blood serum samples collected from five individual mice and recorded the serum volume collected from each mouse. Then, we aliquoted mouse serum into 50, 100 and 200 µL samples for a total of 15 samples used for isolation of EVs. After isolation, EVs were resuspended in 1 mL PBS to measure both number and size of all the vesicles by Nanosight technology (model NS300, Malvern Instruments Ltd., Worcestershire, UK) as described previously [18]. The average values of number and size were acquired from the three starting volumes for each of the 5 mice.

#### 2.2.2. Protein Immunoblot Analysis of EVs

To verify EV marker proteins by immunoblotting [17], we combined the serum from 5 mice in total to generate 1 EV protein sample. The initial EV pellet (purified as above) was resuspended in 20 mL PBS and centrifuged again at 100,000× *g* for 60 min at 4 °C to eliminate the contamination of serum protein. After discarding the supernatant, total EV protein was extracted using 40 µL ice-cold radioimmunoprecipitation assay (RIPA) buffer with protease inhibitor (Sigma-Aldrich, St. Louis, MI, USA) and phosphatase inhibitor (Sigma-Aldrich), and protein concentration was measured by the BCA method (Pierce, Appleton, WI, USA). For immunoblotting, the protein sample was loaded on 4–20% Tris-Glycine precast gels (Bio-Rad, Hercules, CA, USA) and transferred to nitrocellulose membrane. Primary antibodies were diluted in Odyssey blocking buffer (Li-Cor Biosciences, Lincoln, NE, USA) and incubations were performed overnight at 4 °C. Primary antibodies were obtained from the following vendors: Flotillin (Cell signaling, Danvers, MA, USA) and HSP70 (Abcam, Cambridge, UK). Visualization of immunoblot signals was performed using secondary antibodies coupled to Alexa Fluor 680 (Invitrogen Molecular Probes, Eugene, OR, USA) or IRDye 800 (LI-COR Biosciences) and imaged using the Odyssey CLx infrared imager (LI-COR Biosciences). Odyssey version 1.2 imaging software was used to process the images.

#### 2.2.3. Identification of miRNAs in EVs by Reverse Transcriptase-Quantitative Polymerase Chain

##### Reaction (RT-qPCR)

We collected 5 blood serum samples from 5 mice and used the whole serum from individual mice to isolate EVs as described above. We extracted total RNA including miRs from 5 EV pellets individually using the miRNeasy kit (Qiagen, Hilden, Germany) according to the manufacturer’s instructions with minor modification. Briefly, we added 1.5 volume of 100% EtOH to precipitate the total RNA after chloroform cleaning and eluted the RNA using 13 µL water; 1 µL of the RNA sample was used to measure RNA quality and concentration by Nanodrop (Thermo Fisher Scientific, Waltham, MA, USA) and 12 µL of RNA was used to make cDNA by miScript II RT kit, HiFlex buffer (Qiagen). For qPCR, 20 uL of cDNA was diluted by the addition of 200 uL of water. This diluted cDNA was used to evaluate miRs by RT-*q*PCR using miRScript SYBR kit (Qiagen) according to the manufacturer’s instructions, using the CFX96 Touch System (Bio-Rad) and the thermal profile suggested by the manufacturer. Expression levels of miR-21-5p and -16-5p were quantitatively compared using the ∆C_t_ method with mean Ct values of SNORD95, SNORD96 and RNU6-2 as reference genes. These data prove that circulating EVs isolated from a single mouse are enough to evaluate small RNAs.

### 2.3. Sequencing of Small Noncoding RNA Isolated from Mouse Blood EVs with ISO and PE Infusion

#### 2.3.1. Isoproterenol and Phenylephrine Infusion Mouse Model

To carry out small ncRNA NGS in blood EVs, 9 weeks-old male C57/BL6 mice (The Jackson Laboratory) were divided into three groups PBS (*n* = 3), Iso (10 mg/kg/day, *n* = 3) and PE (30 mg/kg/day, *n* = 3), with ISO and PE being infused using osmotic pumps (Alzet, MODEL 1007D) for one week. The ISO was dissolved in 0.002% ascorbic acid in PBS and PE in PBS. The PBS control also contained 0.002% ascorbic acid. On the 6th day. we evaluated heart function and hypertrophy using echocardiography, and at 7 days, mice were euthanized and EVs isolated from blood serum.

#### 2.3.2. Sequencing of Small Noncoding RNA Isolated from Mouse Blood EVs with ISO and PE Infusion

The RNA samples, which were prepared from PBS, ISO and PE infusion mouse blood EVs as described above, were sent to Novogene, Inc., Sacramento, CA, USA for NGS of ncRNA. Novogene did the qualification of the RNA, manufacture of cDNA, the sequence read, data cleaning and data analysis. For the RNA concentration and purity, measurements were done using the NanoDrop 2000 Spectrophotometer (Thermo Fisher Scientific, Wilmington, DE, USA). RNA integrity and quantitation were assessed by Novogene using the RNA Nano 6000 Assay kit and the Agilent Bioanalyzer 2100 System (Agilent Technologies, CA, USA). As discussed in Figure S2B, the input material for the small RNA library and RNA library preparation was performed by Novogene using Multiplex Small RNA Library Prep Set for Illumina (NEB, Ipswich, MA, USA). The library preparations were sequenced on the Illumina platform (NEB, USA) and 50 bp single-end reads were generated.

Sequencing Data Analysis and the clustering of the index-coded samples was performed on a cBot Cluster Generation System using TruSeq SR Cluster kit v3-cBot-HS (Illumina, San Diego, CA, USA). After cluster generation, the libraries were sequenced and 50 bp single-end reads were generated. Clean data were obtained by processing raw data in FASTQ format through custom perl and python scripts. Clean reads less than 50 bp were mapped to reference sequences using Bowtie from miRBase (Release 20) and NCBI mouse genome reference sequences. miREvo and mirdeep2 software was used by Novogene to predict novel miR from the clean data. The DESeq R package (version 1.8.3) was used to identify differential expression of miRs between two groups. The p-values were adjusted using the Benjamin and Hochberg method. A corrected p-value of 0.05 was set as the threshold for significantly differential expression by default. To make the sequencing profiles comparable, the RNA profiles were normalized as read count of a target RNA per million mapped reads (RPM).

#### 2.3.3. Echocardiography

Echocardiography was performed using the Vevo 2100 imaging system from Visual Sonics as described [19]. Briefly, two-dimensional echocardiographic views of the midventricular short axis were obtained at the level of the papillary muscle tips below the mitral valve. M-mode measurements were determined at the plane bisecting the papillary muscles according to the American Society of Echocardiography’s leading-edge method.

### 2.4. Statistics

For experiments in mice, results are presented as mean ±SEM computed from the average measurement obtained from each of the 3 replicates. Comparison of 3 or more groups is performed by one-way ANOVA with Tukey’s multiple comparisons test or non-paired *t*-test. *p* < 0.05 is considered statistically significant. Statistical analysis was performed using GraphPad prism version 8.0.

## 3. Results

### 3.1. Basal Characterization of Circulating EVs from Mouse Blood

**Figure 1:**
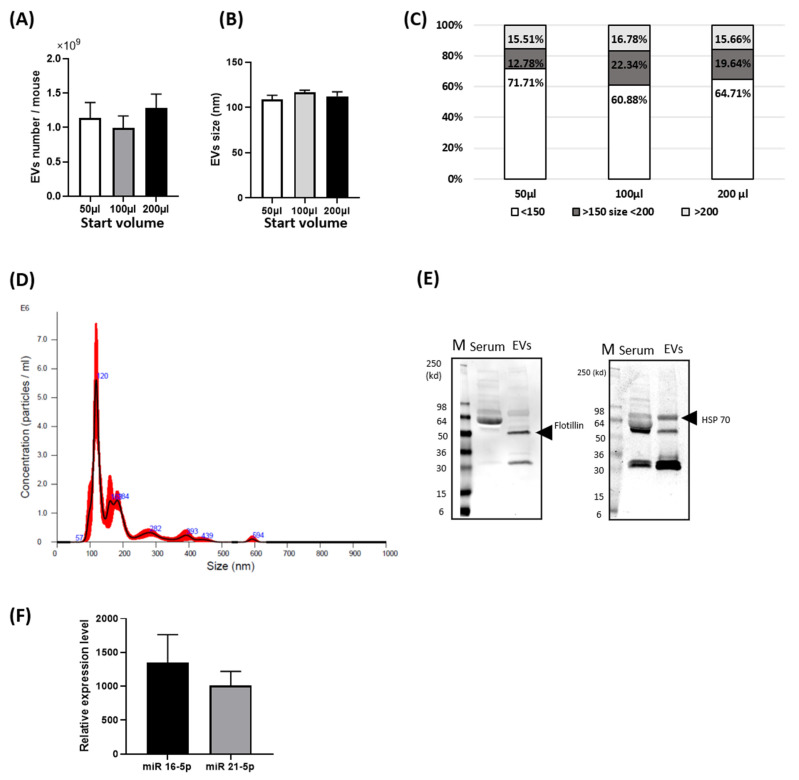
The basal characterization of mouse blood serum extracellular vesicle (EV) and verification of EVs miRNA: (**A**) Bar graph of EVs’ number measured by Nanosight in three different start serum volumes, 50 µL, 100 µL and 200 µL of mouse serum (*n* = 5); (**B**) The bar graph of average exosome size measured in three different start serum volumes of mouse serum; (**C**) Bar graph of distribution of EVs’ size in three different start serum volumes of mouse serum. EVs’ size are categorized by less than 150 nm, between 150 to 200 and over 200 nm; (**D**) Representative histogram of EVs’ size distribution measured at 100 µL starting serum volume by Nanosight; (**E**) Immunoblot of five mice blood EVs’ pooling protein sample by anti-HSP70 Ab and anti-Flotillin Ab as EVs’ marker protein. Serum line loads same mouse blood serum protein amount as EVs’ sample; (**F**) Bar graph of relative expression level of miRNA (miR)-21-5p and miR-16-5p in mouse blood EVs measured by real-time PCR. The expression levels of two miRs were normalized by SNORD95, SNORD96A, RNU6-2 by RT-PCR. (*n* = 5). Mean ± SE.

For basal characterization of blood EVs, as shown in Figure 1, we first validated the presence of the EVs from mouse serum using Nanosight spectrometry. The average mouse body weight used for blood EV purification and size analysis was 25.4 ± 2.4 g (*n* = 5) and total blood serum volume 459.0 ± 72.2 µL (*n* = 5). The number of EVs found in mouse serum was 1.01 × 10^9^ ± 4.16 × 10^8^/mouse and the average size was 116.6 ± 4.8 nm (Figure 1A,B) using 100 µL starting volume. The majority of EV size was found to be less than 150 nm (Figure 1C,D) and these values were consistently found when 3 different volumes were loaded onto Nanosight (50, 100 and 200 μL).

HSP70 and Flotillin are accepted as markers of EVs/exosomes [20] and we found expression of both proteins in our EV samples examined by immunoblotting. As shown in Figure 1E, these proteins were robustly expressed in blood serum EVs and not detectable in the same amount of blood serum protein. To confirm the presence of miR in blood serum EVs, we measured the expression level of two miRs by RT-PCR. The miR-21-5p and miR-16-5p were detected similarly in five mice blood serum EVs’ samples (Figure 1F). The concentration and quality of total RNA of EVs and the Ct values of RT-PCR were provided as Tables S1 and S2.

### 3.2. Phenotypic Characterization of Mice after Chronic Infusion of PE or ISO

Seven days of ISO or PE infusion into healthy mice resulted in increased wall thickness as measured by echocardiography compared to PBS-treated control mice (Figure S1A,B). These changes were found during systole and diastole. Interestingly, PE, but not ISO, significantly decreased ventricular dimensions (Figure S1C). Cardiac function as determined by echocardiography was increased after 7 days of both ISO and PE (Figure S1D,E) with only ISO increasing heart rate as would be expected after β-adrenergic stimulation (Figure S1F). Both ISO and PE also increased heart size compared to PBS-treated controls as determined by either the ratio of heart weight to body weight or to tibia length determined after sacrifice (Figure S1G).

### 3.3. Quality Evaluation of Total RNA of Serum EVs

The concentration and size distribution of total RNA in blood EVs were determined with an Agilent Bioanalyzer 2100. The RNA of serum EVs from PBS-, ISO- and PE-treated mice distributed in less than 500 nucleotides (nt) with a majority under 200 nt without visible 18S and 28S peaks (Figure S2A). The total RNA amount in EVs in the 3 groups (ISO, PE and PBS) ranged from 1.29 ng to 5.7 ng (Figure S2B) and all of the total RNA amount was used for cDNA library preparation.

### 3.4. Comparison of Small ncRNA Content of Serum EVs

**Figure 2:**
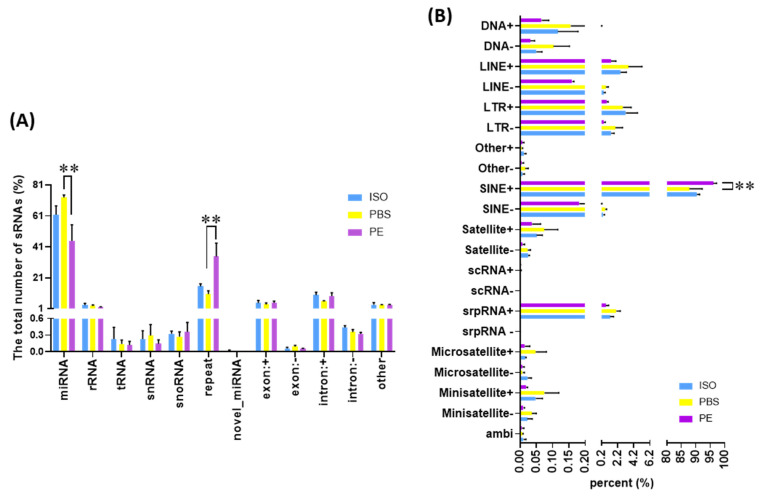
The frequency of annotated reads of small RNAs and the classes of genomic repeat in mouse blood extracellular vesicles (EVs) measured by noncoding small RNA sequencing: (**A**) Bar graph of the annotated small RNA species and their percentages in total read mapped to genome in mouse blood EVs with PBS, ISO (10 mg/kg/day) and PE (30 mg/kg/day) infusion for 1 week; (**B**) Bar graph of the classes of genomic repeat and their percent. PBS (*n* = 3), ISO (*n* = 3) and PE (*n* = 3). Mean ± SD. ** *p* < 0.01.

Small RNA sequencing was performed to elaborate the differences in RNA species of the different serum EVs collected. According to the annotated data, blood EVs analyzed contained miRNA, rRNA, tRNA, snRNA, snoRNA, repeat, exon: +, exon: − intron: +, intron: − and other reads (Figure 2A). As shown in Figure 2A, the majority of RNA reads consisted of miRs. Interestingly, EVs from PE-treated mice had significantly decreased miR content compared to EVs from ISO-treated mice while PE caused increased repeat elements compared to both ISO and PBS (Figure 2A). As shown in Figure 2B, the SINE + among repeat classes was increased significantly by PE.

### 3.5. Differentially Expressed miRs in Different Serum EVs

**Figure 3:**
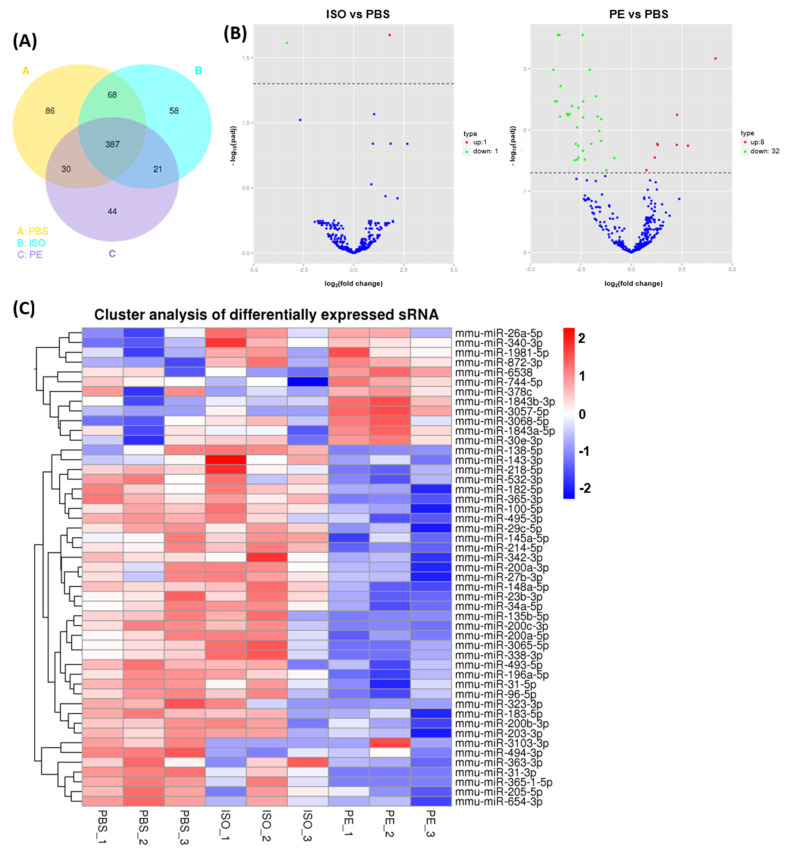
The analysis of differentially expressed miRNAs and cluster analysis by noncoding small RNA next generation sequencing in mouse blood serum extracellular vesicle (EV): (**A**) Venn diagram representing the presence, absence, or overlap of miRNAs identified through NGS in the blood EVs of PBS, ISO and PE infusion mouse; (**B**) The volcano chart of differential expressed miRNAs in ISO versus PBS and PE versus PBS; (**C**) Heat map of hierarchical clustering of differentially expressed miRNAs. PBS (*n* = 3), ISO (*n* = 3, 10 mg/kg/day) and PE (*n* = 3, 30 mg/kg/day) for 1 week.

NGS identified 849 known miRs and 37 novel miRs. Out of these, 694 miRs were identified in EV samples (3 groups of *n* = 3 each—PBS-, ISO- and PE-treated mice) and 387 miRs were found to be commonly identified in all 3 groups (Figure 3A). As shown in Figure 3A, unique miRs were found in each group as well as with 86 unique miRs in PBS- treated mice, 58 miRs in ISO-treated mice and 44 miRs in the PE-treated mice. Shown in Figure 3B are volcano plots for differential miR expressed in EVs from ISO vs. PBS mice and PE vs. PBS mice with multiple miRs with significant differences between the groups. Specifically, PE caused the up-regulation of 8 miRs and down-regulation of 36 miRs in serum EVs compared to the content of EVs isolated from the serum of PBS-treated mice (Table 1 and Table 2). Among those miRs up-regulated by PE, miR-26a-5p was the most abundant miR in blood EVs and also had the biggest change at read count number. Interestingly, ISO caused serum EVs to have only a single up-regulated miR, miR-340-3p, and miR-3103-3p was the single down-regulated miR when compared to blood EVs from PBS- treated mice (Table 3). Interestingly, both miR-26a-5p and -340-3p, up-regulated by PE and -340-3p up-regulated by ISO was positioned in the same cluster in cluster analysis (Figure 3C). Those two miRs may be a common and a major target altered by AR-receptor stimulation.

### 3.6. Functional Pathway Analysis of Differentially Expressed Serum EV miRs

**Figure 4:**
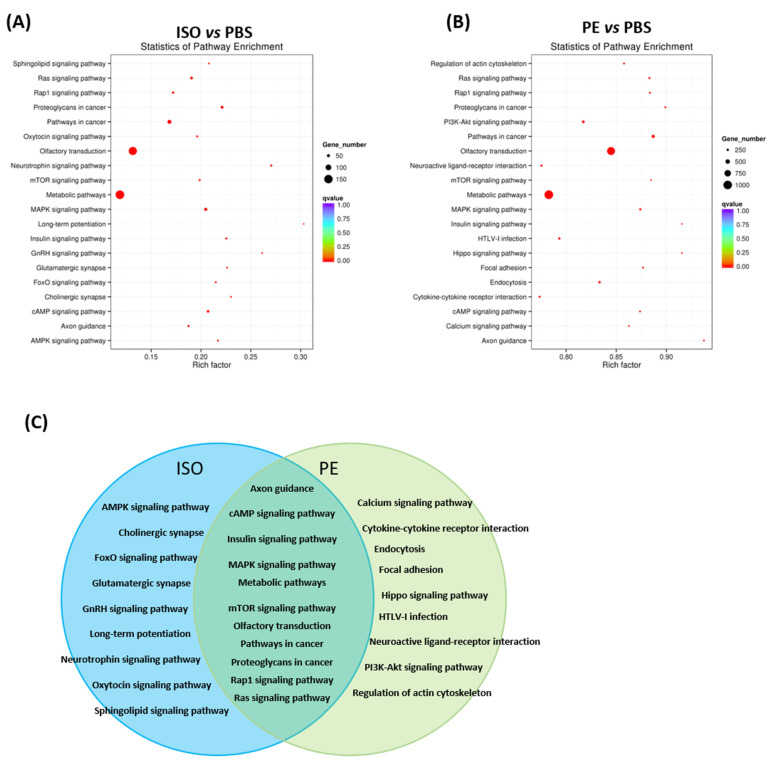
Differentially expressed miRNAs target mRNA genes in enriched KEGG pathway: (**A**,**B**) Scatter plot KEGG pathway enrichment statics; (**A**) ISO versus PBS; (**B**) PE versus PBS; (**C**) Venn diagrams showed 11 overlap pathways and 9 different pathways detected in ISO and PE blood EVs. PBS (*n* = 3), ISO (*n* = 3, 10 mg/kg/day) and PE (*n* = 3, 30 mg/kg/day) infusion for 1 week.

The top 20 Kyoto Encyclopedia of Genes and Genomes (KEGG) pathways were identified via enrichment analysis using differentially expressed miRs (Figure 4A,B). ISO and PE shared 11 pathways with 9 each un-shared and unique (Figure 4C). Among the 11 overlapping pathways between ISO and PE were cAMP signaling, insulin signaling and MAP Kinase signaling (Figure 4C). ISO caused enrichment of miRs involved in AMPK signaling, FOXO signaling and neurotrophin signaling among others, while PE specifically enriched miRs associated with calcium signaling, Hippo signaling and PI3-Akt signaling pathway and others (Figure 4C), cytokine-cytokine receptor interaction, endocytosis, focal adhesion, HTLV-I infection and neuroactive ligand-receptor interaction and regulation of actin cytoskeleton.

## 4. Discussion

In this study, we have demonstrated for the first time, how chronic stimulation of αAR and βAR systems, using 7 days of treatment with PE and ISO, respectively, causes differential and specific loading of ncRNAs in circulating EVs. Importantly, both ISO and PE administration caused unique enrichment of miRs when compared to PBS-treated control C57B/6 mice. The model used was to designed to mimic chronic sympathetic nervous system activity found in cardiovascular disorders such as heart failure when circulating catecholamines are high, over-stimulating ARs.

Interestingly, PE treatment increased the read counts of genomic repeat elements found in serum EVs, in particular, SINE + elements. This repeat RNA can cause toxicity by directly interacting with repeat RNA-binding proteins and thereby compromising their normal function [21]. This is referred to as “RNA toxicity” [22]. This repeat RNA might also induce toxicity indirectly by being translated into toxic proteins [23]. The up-regulation of genomic repeat regions in blood EVs by PE might be a reason why EVs made during disease processes could be pathogenic to recipient cells. Thus, EV cargo made during different cardiovascular disease (CVD) may be used as a biomarker and also potentially a therapeutic target to limit these damaging genomic repeat elements.

Overall, PE caused the most changes in ncRNA cargo. One of the most up-regulated miRs was mmu-miR-26a-5p, that is robustly expressed in control EVs as well but still significantly enhanced after PE. Recently, miR26a was found in both EVs from body fluids and cell-secreting EVs. Muscle satellite cells secreting miR26a-rich exosome attenuated the wasting of skeletal muscle and the cardiac fibrosis of chronic kidney disease mouse [24]. Fumihiko et al. showed that miR-26a regulated EVs’ secretion from cancer cell via targeting SHC4, PFDN4 and CHORDC1. miR-26a mimic transfection decreased the exosome secretion [25]. These results suggest that miR-26a may correlate with EV biogenesis and also affects inner cellular mechanisms. ISO up-regulated only one miR-340-3p. Although this miR appears to have a role in regulating genes in cancer biology, its role in myocytes has not been investigated.

As shown in Table S3, we extracted the most abundant 10 miRs in PBS group in total read count and in transcripts per million (TPM). There are marked mmu-miR-21a-5p, as well as mmu-miR-486a-3p, -5p and -486b-3p and -451a. Those five miRs composed almost 50% of TPM in each group, which is very impressive. In regards to the significance of regulating miR transcription, miR copy number is one major factor as well as differential expression found in the different EVs. Really abundant miRs have the potential to be real effectors to cells that take up circulating EVs. Of the five most abundant miRs, three were decreased in ISO- or PE-treated circulating EVs. Probably, the five miRs would be a major effector to recipient cells and in particular, three miR-486a and -486b were decreased in ISO at TPM result and in both ISO and PE at read count.

The amount of erythrocyte-derived miR represents the majority of miRs expressed in whole blood. miR-451, miR-144, and miR-486 (mmu-486a and -486b), which are abundant in red blood cells (RBCs), are involved in the process of erythropoiesis and disease occurrence [26]. One of them has miR486-5p (mmu-486a-5p); Li-Shen et al. reported the miR- 486-5p regulate normal erythropoiesis and growth [27]. Extensive evidence has shown that miR-451 (mmu-miR-451a) and miR-144 are crucial to erythropoiesis [28,29]. Furthermore, β-adrenergic mechanisms regulate erythropoiesis in erythropoietin-resistant anemia [30] and in resultant anemia after severe injury [31]. Those previous and our current results suggest that the majority of blood EVs could be from red blood cells and both AR stimulation affected RBC-related miRs found in circulating EVs.

KEGG functional and pathway enrichment analysis of the target genes predicted by differentially expressed miRs by ISO and PE was performed to understand the screened target genes better. We found 11 pathways shared another 9 pathways for each distinct AR stimulation. Among these pathways affected were several neurotransmitter or synapse pathways and calcium/cAMP signaling pathways. ISO showed three nerve synaptic pathway, in particular the ‘long-term potentiation’ with highest rich factor being a special feature of βAR for heart dysfunction and brain memory disorder [32]. The PI3-Akt/mTOR/calcium signaling pathway can be a major pathway downstream of both ARs and in situations of crosstalk between α-AR and β-AR, these signaling pathways appear more dominant after PE-mediated αAR stimulation.

Importantly, this study demonstrates that technically, the blood of a single mouse is sufficient for EV isolation and for subsequent ncRNA NGS. Overall, the amount of EVs from a single mouse was low compared to another report using the Exo Quick method (System Biosciences, Inc., Palo Alto, CA, USA) [14]. Further, the number of EVs was reported in rat plasma purified via ultra-centrifugation to be 1 × 10^10^/mL [33] and as high as 2.5 × 10^12^/mL isolated by Exo Quick [14]. Thus, our results are close to what was reported for rat plasma. A limitation of our study is a large individual mouse variance in EV number and a change in the initial starting serum volume in our analysis did not reduce the variance (Figure 1A).

According to a paper that shared information on the concentration of exosome/EV RNA used for NGS, the minimum amount is 2–2.5ng [34,35] and some of them used up to 100 ng [13], which has been stated as the ideal amount to make a cDNA library in both human [13,34] and rodent blood [23]. In our study, using 1.29–5.71 ng RNA, a total of 886 miRs (849 known and 37 novel) were identified. For the control PBS-treated mouse group, the number of miRs classed by tags per million (TPM) interval were 438 miRs where TPM interval 0–3.57; the 139 miRs where TPM interval 3.57–15; the 117 miRs where TPM interval 15–60 and the 192 microRNAs where TPM > 60. In a recent study, human blood EVs and NGS study with the same analysis platform of ours, revealed 345 unique microRNA where TPM ≥ 1 and 164 unique microRNAs where TPM ≥ 10 [36].

Our PE and ISO mouse model showed, after 7 days of treatment, significant cardiac hypertrophy but no significant ventricular dysfunction. Perhaps longer treatment times that do cause HF may lead to more dramatical changes or larger differential data sets and could be a future study. However, our results did show more dramatic results with PE-treatment compared to ISO, which is of interest. This is somewhat surprising since catecholamines that are elevated in cardiovascular disorders are thought to affect myocardial βARs more than αARs, which are found more in vascular smooth muscle than cardiac tissue. In fact, chronic βAR stimulation has always been more strongly linked to disease progression. This is interesting since exosome/EV biogenesis and miR-mediated gene silencing may be functionally linked [3,37,38] and thus, chronic βAR stimulation from circulating catecholamines may alter how EVs could bring harmful components to distant recipient cells.

## 5. Conclusions

Both α- and β-AR agonists can alter small RNA content of circulating blood EVs/exosomes including differential expression and loading of miRs that indicate regulation of distinct pathways. This study provides novel insight into chronic sympathetic nervous system activation in HF where excessive catecholamine may not only participate in pathological remodeling of the heart but alter other organs due to circulating EVs.

## Figures and Tables

**Table 1:** miRNAs with significant up-regulation in blood serum extracellular vesicles (EVs) with PE (30 mg/kg/day) infusion for 1 week versus PBS.

miRNA	PE (Read Count)	PBS (Read Count)	log2FoldChange
mmu-miR-3057-5p	27.55	0.19	4.16
mmu-miR-872-3p	33.03	2.29	2.80
mmu-miR-1843b-3p	115.24	19.78	2.26
mmu-miR-3068-5p	182.56	30.11	2.25
mmu-miR-1981-5p	795.19	309.24	1.30
mmu-miR-340-3p	191.34	76.10	1.28
mmu-miR-30e-3p	781.70	339.02	1.16
mmu-miR-26a-5p	41,301.67	24,470.54	0.74

**Table 2:** miRNAs with significant down-regulation in blood serum EVs with PE (30 mg/kg/day) infusion for 1 week versus PBS.

RNA	PE (Read Count)	PBS (Read Count)	log2FoldChange
mmu-miR-365-3p	0.90	52.51	−3.85
mmu-miR-365-1-5p	0.00	29.88	−3.77
mmu-miR-214-5p	0.52	43.74	−3.70
mmu-miR-200a-5p	8.86	189.11	−3.64
mmu-miR-205-5p	421.15	8177.68	−3.56
mmu-miR-31-3p	0.00	22.30	−3.53
mmu-miR-323-3p	1.79	45.17	−3.50
mmu-miR-135b-5p	1.79	34.68	−3.18
mmu-miR-654-3p	2.69	53.94	−3.13
mmu-miR-494-3p	2.83	46.99	−3.05
mmu-miR-34a-5p	7.50	91.43	−2.93
mmu-miR-532-3p	2.16	43.68	−2.81
mmu-miR-196a-5p	7.01	81.23	−2.75
mmu-miR-96-5p	21.56	206.15	−2.69
mmu-miR-493-5p	4.19	56.35	−2.68
mmu-miR-495-3p	4.44	49.01	−2.63
mmu-miR-148a-5p	7.88	67.58	−2.62
mmu-miR-29c-5p	3.73	45.12	−2.61
mmu-miR-200c-3p	576.38	3452.40	−2.41
mmu-miR-31-5p	43.08	322.11	−2.40
mmu-miR-183-5p	274.95	1734.91	−2.38
mmu-miR-218-5p	70.60	459.62	−2.38
mmu-miR-145a-5p	98.67	692.59	−2.30
mmu-miR-200b-3p	768.80	3522.19	−2.06
mmu-miR-363-3p	355.44	1637.29	−1.98
mmu-miR-203-3p	886.93	3145.32	−1.74
mmu-miR-182-5p	205.97	706.32	−1.68
mmu-miR-100-5p	2259.26	7238.42	−1.59
mmu-miR-23b-3p	216.79	646.32	−1.49
mmu-miR-200a-3p	576.49	1708.26	−1.49
mmu-miR-342-3p	74.86	185.36	−1.24
mmu-miR-27b-3p	4002.02	7246.92	−0.84

**Table 3:** miRNAs with significant up and down-regulation in blood serum EVs with ISO (10 mg/kg/day) infusion for 1 week versus PBS.

miRNA	ISO	PBS	log2FoldChange
mmu-miR-340-3p	320.51	85.06	1.79
mmu-miR-3103-3p	0.00	20.73	−3.32

## Data Availability

In accordance with MDPI Research Data Policies, we intend to upload raw NGS data to Gene Expression Omnibus (GEO) if the manuscript is accepted for publication.

## Supplementary Materials

The following are available online at https://www.mdpi.com/article/10.3390/cells10051211/s1, Figure S1: Basal characteristics of PBS, ISO and PE infusion mouse model to small RAN sequencing in blood extracellular vesicle (EV), Figure S2: The length distribution and concentration of small RNAs in mouse blood EV samples as detected by Agilent 2100 Bioanalyzer using small RNA chips, Table S1: Qualification of total RNA purified from blood serum EVs for basal characterization of blood EVs, Table S2: Ct values of SNORD95, SNORD96A, RNU6-2, miR 21-5p, miR 16-5p and GAPDH of in mouse blood EVs measured by real time-PCR for basal characterization of blood EVs, Table S3: Expression of the top 10 miRNAs in blood EV of mice with PBS, ISO and PE injection for 1 week. The read count and TPM values provided here are the average values of three biological replicates.
