# Supplementary material for: In Vivo Stimulation of α- and β-Adrenoceptors in Mice Differentially Alters Small RNA Content of Circulating Extracellular Vesicles
Source: Cells. 2021 May 15;10(5):1211. doi: 10.3390/cells10051211 (PMC8156306; doi:10.3390/cells10051211)
Supplement: Supplementary file 1 [file cells-10-01211-s001.zip › cells-1206207-supplementary.pdf]

Supplementary

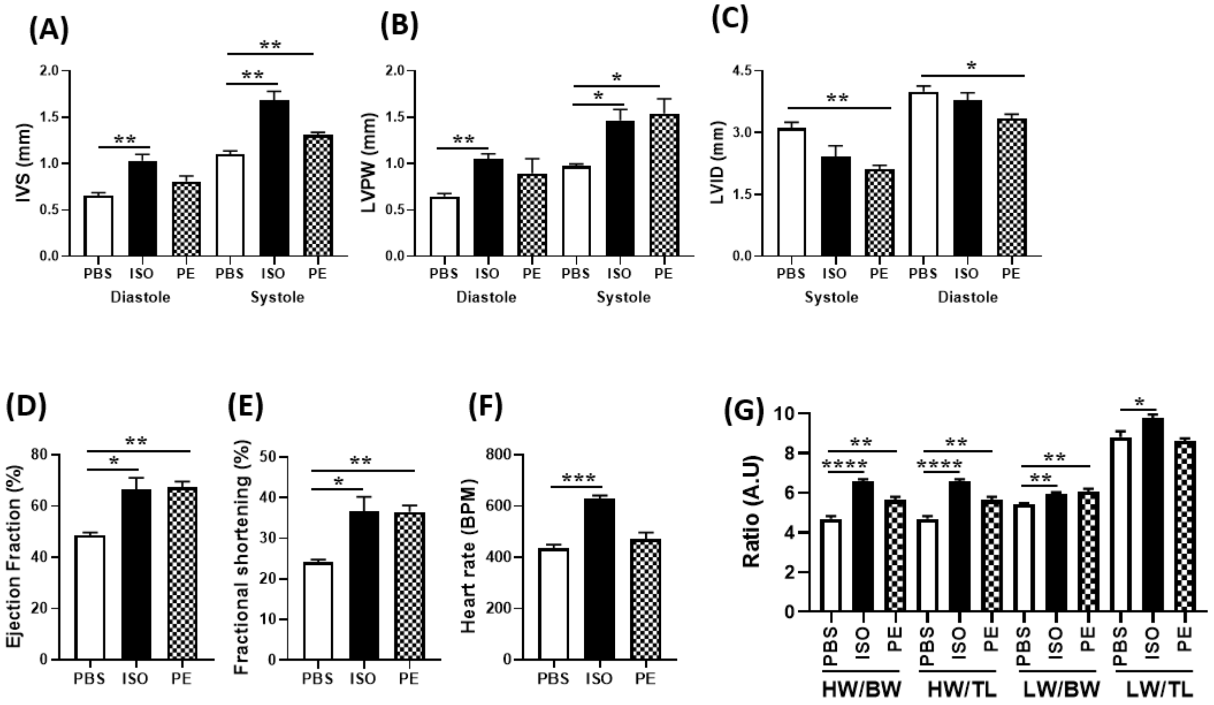

Figure S1. Basal characteristics of PBS, ISO and PE infusion mouse model to small RAN sequencing in blood extracellular vesicle (EV). A to F. Parameters of heart function measured by echocardiology. (**A**) Bar graph of Interventricular septal (IVS) end diastole and end systole (**B**) Left ventricular posterior wall (LVPW) end diastole and end systole. (**C**) Left ventricular internal diameter (LVID) end diastole and end systole. (**D**) and (**E**) Bar graph of Ejection fraction and Fraction shortening. (**F**) Bar graph of heart rate/min. (**G**) The ratio of heart weight (HW, mg) with body weight (BW, g) and tibia length (TL, mm). Mean ± SE. PBS (*n* = 3), ISO (10 mg/kg/day, *n* = 3) and PE (3 omg/kg/day, *n* = 3) infusion for 1 week. Mean ± SE, * p < 0.05, ** p < 0.01, *** p < 0.005, **** p < 0.001.

(A)

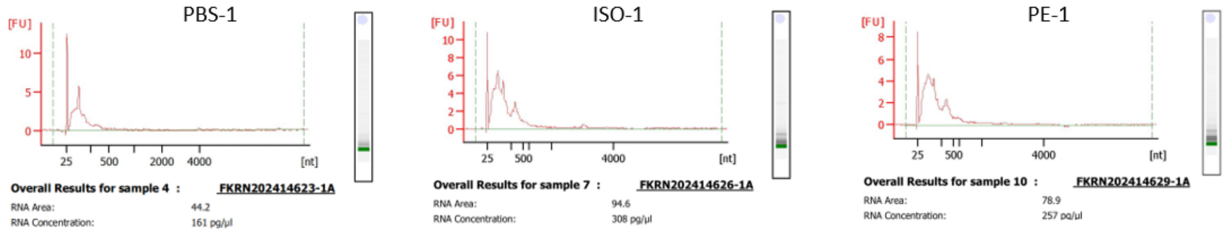

(B)

Name	Concentration (ng/ul)	Volume (ul)	Amounts (ug)
**PBS 1**	0.161	8	0.00129
**PBS 2**	0.175	9	0.00158
**PBS 3**	0.289	8	0.00231
**ISO 1**	0.308	9	0.00271
**ISO 2**	0.32	11	0.00352
**ISO 3**	0.432	11	0.00475
**PE 1**	0.257	9	0.00231
**PE 2**	0.381	9	000343
**PE 3**	0.714	8	0.00571

Figure S2. The length distribution and concentration of small RNAs in mouse blood EVs samples as detected by Agilent 2100 Bioanalyzer using small RNA chips. (**A**) Representative electropherograms and gel-like image of total RNA of mouse blood EVs with PBS, ISO (10 mg/kg/day) and PE (30 mg/kg/day) infusion for 1 week. (**B**) Table of the concentration of small RNA (25–200 nt) in each mouse blood EVs with PBS, ISO and PE and the amounts of RNA used to make cDNA library. PBS (n = 3), ISO (n = 3) and PE (n = 3).

Table S1. Qualification of total RNA purified from blood serum EVs for basal characterization of blood EVs. (n = 5)

Mouse ID	Concentration (ng/μL)	260/280 (Ratio)	260/230 (Ratio)
# 6	10	1.41	0.51
# 7	12	1.49	0.06
# 8	14	1.4	0.22
# 9	9.3	1.43	0.26
# 10	17.4	1.45	0.04
Ave	12.54	1.44	0.22
STDEV	3.28	0.04	0.19

Table S2. Ct values of SNORD95, SNORD96A, RNU6-2, miR 21-5p, miR 16-5p and GAPDH of in mouse blood EVs measured by real time-PCR for basal characterization of blood EVs. (n = 5).

Mouse ID	Ct Value of Non-Coding Small RNAs and GAPDH by RT-PCR						
	SNORD95	SNORD96A	RNU6-2	Ave Ct	miR21-5p	miR16-5p	GAPDH
# 6	28.27	33.36	33.66	31.76	21.24	20.28	25.83
# 7	29.33	33.35	34.93	32.54	22.60	23.24	25.95
# 8	28.47	33.03	33.75	31.75	23.66	21.29	26.41
# 9	29.86	34.05	36.49	33.46	23.13	23.23	27.00
# 10	29.85	34.53	35.40	33.26	23.21	23.92	26.44
Ave	29.15	33.67	34.84	32.55	22.77	22.39	26.33
STDEV	0.75	0.61	1.19	0.81	0.93	1.54	0.46

Table S3. Expression of the top 10 miRNAs in blood EV of mice with PBS, ISO and PE injection for 1 week. The Readcount and TPM values provided here are the average values of three biological replicates. (*n* = 3)

	**Readcount (ave)**			**TPM (ave)**		
	**ISO**	**PBS**	**PE**	**ISO**	**PBS**	**PE**
mmu-miR-451a	300,671	397,360	61,577	160,589	119,593	58,788
mmu-miR-486a-3p	107,312	377,999	123,033	71,364	109,307	116,475
mmu-miR-486a-5p	107,177	376,634	122,004	71,226	108,954	115,469
mmu-miR-486b-3p	106,416	374,515	121,170	70,714	108,335	114,671
mmu-miR-21a-5p	213,110	276,566	86,902	138,434	80,236	78,239
mmu-let-7i-5p	25,864	110,784	15,520	20,014	32,735	14,657
mmu-miR-92a-3p	15,471	72,343	17,649	11,723	20,776	16,483
mmu-miR-148a-3p	39,966	64,012	16,731	27,613	19,836	15,041
mmu-let-7f-5p	19,890	58,817	13,553	15,067	17,737	12,879
mmu-let-7a-5p	14,270	55,007	9,933	11,116	17,148	9,425
